# Supplementary material for: Dynamic 18F-Pretomanid PET imaging in animal models of TB meningitis and human studies
Source: Nat Commun. 2022 Dec 29;13:7974. doi: 10.1038/s41467-022-35730-3 (PMC9800570; doi:10.1038/s41467-022-35730-3)
Supplement: Supplementary file 1 — Supplementary Information file [file 41467_2022_35730_MOESM1_ESM.docx]

Dynamic ^18^F-Pretomanid PET imaging in animal models of TB meningitis and human studies

**Authors:** Filipa Mota^1,2,3†^, Camilo A. Ruiz-Bedoya^1,2,3†^, Elizabeth W. Tucker^1,2,4†^, Daniel P. Holt^5†^, Patricia De Jesus^1,2,3^, Martin A. Lodge^5^, Clara Erice^1,2,4^, Xueyi Chen^1,2,3^, Melissa Bahr^1,2,3^, Kelly Flavahan^1,2,3^, John Kim^1,2,4^, Mary Katherine Brosnan^5^, Alvaro A. Ordonez^1,2,3^, Charles A. Peloquin^6^, Robert F. Dannals^5^, Sanjay K. Jain^1,2,3,5*^

**Affiliations:**

^1^Center for Infection and Inflammation Imaging Research, Johns Hopkins University School of Medicine; Baltimore, MD 21287, USA

^2^Center for Tuberculosis Research, Johns Hopkins University School of Medicine; Baltimore, MD 21287, USA

^3^Department of Pediatrics, Johns Hopkins University School of Medicine; Baltimore, MD 21287, USA

^4^Department of Anesthesiology and Critical Care Medicine, Johns Hopkins University School of Medicine; Baltimore, MD 21287, USA

^5^Russell H. Morgan Department of Radiology and Radiological Sciences, Johns Hopkins University School of Medicine; Baltimore, MD 21287, USA

^6^Infectious Disease Pharmacokinetics Laboratory, Pharmacotherapy and Translational Research, University of Florida College of Pharmacy; Gainesville, FL 32610, USA

^†^Authors contributed equally

^*^Corresponding author**.** Email: [sjain5@jhmi.edu](mailto:sjain5@jhmi.edu)

**METHODS**

**General Materials:** No unexpected or unusually high safety hazards were encountered. All chemicals and solvents were reagent grade and purchased from a commercial supplier. They were used as received unless otherwise noted. The syntheses of the radiolabeling precursor and intermediates were performed by Axia Chemicals Ltd. (Kent, U.K.). Whenever required, reactions were conducted under a nitrogen atmosphere and anhydrous solvents were utilized. Analytical thin layer chromatography was performed with Merck silica gel 60 F254 plates. Chemical shifts (δ) are reported in parts per million (ppm) relative to residual undeuterated solvent as the internal reference and coupling constants (*J*) are reported in Hertz (Hz). Splitting patterns are indicated as follows: s = singlet, d = doublet, t = triplet, q = quartet, qn = quintet, dd = doublet of doublet, dt = doublet of triplet, m = multiplet, br = broad peak.

**Chemical synthesis of precursors**

A synthetic scheme is represented in Fig. S1.

Ethyl 2,2-difluoro-2-(*p*-tolyloxy)acetate **(3):** *p*-Cresol **1** (36.76 g, 0.34 mol), was dissolved in anhydrous *N,N*-dimethylformamide (350 mL) under argon. The solution was cooled to 10 °C and hexane washed sodium hydride (19.04 g, 1.4 x, 60%) was added portion wise. Once the addition was complete, stirring was continued until effervescence had ceased. Ethyl bromodifluoroacetate **2** (100 g, excess) was added dropwise (neat) over a fairly short period (20 min) with cooling. Considerable foaming was observed. Once the addition was complete, stirring was continued as the mixture warmed to room temperature. The excess sodium hydride was destroyed by the dropwise addition of cold water. The solution was poured into water (3 L) and the aqueous solution was extracted with diethyl ether (2 x 500 mL). The combined extracts were washed with brine and dried over anhydrous magnesium sulphate. The solvent was removed under reduced pressure, and the oil obtained was purified over SiO_2_ eluting with 5% diethyl ether / hexane to yield the product as a colorless mobile oil (44.45 g, 57%). ^1^H NMR (500 MHz, CDCl_3_) δ 7.10 (dd, *J* = 9.5, 7.5 Hz, 4H), 4.35 (q, *J* = 9.0 Hz, 2H), 2.33 (s, 3H), 1.35 (t, *J* = 8.5 Hz, 3H).

2,2-difluoro-2-(*p*-tolyloxy)acetic acid **(4):** The prepared ester **3** (43.74 g, 0.19 mol) was dissolved in acetonitrile (400 mL), and chilled. A cold aqueous solution of sodium hydroxide (200 mL, 5M) was added steadily, though rapidly, and the mixture was stirred for 5 h at room temperature. The volume was reduced *in vacuo*, and the aqueous solution was acidified with cold dilute hydrochloric acid (600 mL, 2M). The oily aqueous solution was extracted with dichloromethane (2 x 400 mL), and the combined extracts were washed with brine, and dried over anhydrous magnesium sulphate. The solvent was evaporated under reduced pressure, and the oil obtained was utilized without further purification (20.53 g, 53%).

1-(bromodifluoromethoxy)-4-methylbenzene **(5):** To a solution of the prepared carboxylic acid **4** (8.09 g, 0.04 mol) dissolved in dichloromethane (80 mL) was added anhydrous *N,N*-dimethylformamide (0.5 mL, catalyst) followed by the dropwise addition of oxalyl chloride (7.62 g, 0.06 mol) at 0°C. The reaction mixture was stirred at room temperature for 3 h, then concentrated *in* *vacuo.* To the prepared acid chloride was added: bromochloromethane (100 g, excess), dimethylaminopyridine (1.22 g, catalyst), and 2-mercaptopyridine *N*-oxide sodium salt (5.97 g, 0.04 mol). The mixture was heated under reflux for 2 h (120 °C, oil bath temperature). On cooling, the excess bromochloromethane was removed under reduced pressure, and the residue was purified over SiO_2_ eluting with 2% diethyl ether / hexane to yield the product as a colorless mobile oil (7.05 g, 74%). ^1^H NMR (500 MHz, CDCl_3_) δ 7.10 (dd, *J* = 8.5, 7.5 Hz, 4H), 2.25 (s, 3H).

1-(bromodifluoromethoxy)-4-(bromomethyl)benzene **(6):** The prepared substrate (3.32 g, 0.14 mol) was dissolved in carbon tetrachloride (50 mL) and *N*-bromosuccinimide (2.49 g, 0.14 mol) and benzoyl peroxide (0.25g, initiator) were added. The mixture was heated under reflux for 5 h. On cooling, the solvent was removed under reduced pressure and the residue was purified over SiO_2_ eluting with 5% diethyl ether / hexane to yield a colorless mobile oil (2.17 g, 49% crude yield). ^1^H NMR (500 MHz, CDCl_3_) δ 7.41 (d, *J* = 9.0 Hz, 2H), 7.20 (d, *J* = 8.50 Hz, 2H), 4.47 (s, 2H).

(*S*)-6-((4-(bromodifluoromethoxy)benzyl)oxy)-2-nitro-6,7-dihydro-5*H*-imidazo[2,1-*b*][1,3]oxazine **(7):** The reactants, the prepared benzyl bromide **6** (2.06 g, 0.0065 mol, 1.3 x), and (*S*)-2-nitro-6,7-dihydro-5H-imidazo[2,1-b][1,3]oxazin-6-ol (0.97 g, 0.005 mol) were dissolved in anhydrous *N,N*-dimethylformamide (30 mL) and cooled to 5°C.  Hexane washed sodium hydride (0.32 g, 0.008 mol, 1.6 x, 60%) was added in portions under an argon atmosphere. Once the addition was complete, the mixture was warmed to room temperature, and stirred for 3 h. The slurry was re-chilled, and carefully treated with water (dropwise), then brine. The mixture was poured into brine (600 mL) and the aqueous solution was extracted with ethyl acetate (5 x 200 mL). The combined extracts were washed with water, brine, and the solvent was dried over anhydrous magnesium sulphate, and subsequently evaporated *in* *vacuo*. The solid obtained was washed with chilled diethyl ether. The crude product was purified over SiO_2_ eluting with dichloromethane (the primary impurity elutes in reverse). At low volume, ethyl acetate was added and evaporation was continued to a very low level. The product was collected, and washed with ethyl acetate at -70°C, then pentane, to yield needles (1.07 g, 51%).^1^H NMR (500 MHz, *d*_6_-DMSO) δ 8.04 (s, 1H), 7.45 (d, J = 8.5 Hz, 2H), 7.35 (d, J = 7.9 Hz, 2H), 4.73 – 4.65 (m, 3H), 4.48 (dd, J = 12.0, 1.0 Hz, 1H), 4.32 – 4.21 (m, 3H). ^13^C NMR (126 MHz, *d*_6_-DMSO) δ 206.96, 148.20, 147.57, 142.56, 137.78, 129.88, 121.44, 119.52, 118.49, 69.19, 68.30, 67.04, 47.22, 31.15. HRMS (ESI+) calculated for [M*+H]^+^ C_14_H_13_N_3_O_5_F_2_Br 420.00, found m/z = 420.0013.

**Radiosynthesis of ^18^F-pretomanid**

*Manual synthesis (silver(I)-mediated):* Aqueous ^18^F-fluoride (2 mL) was trapped on a QMA cartridge pre-equilibrated with Trace-Select Water (10 mL). K^18^F was eluted from the cartridge with a solution of potassium oxalate (4 mg, 24 µmol), potassium carbonate (0.2 mg, 1.4 µmol) and dicyclohexano-18-crown-6 (14 mg, 38 µmol) in a mixture of acetonitrile (80%) and water (1 mL) into a Wheaton v-vial. The solution was dried under a constant stream of N_2_ for 15 min at 110 °C. K^18^F was then dried by azeotropic distillation with acetonitrile (400 µL, twice) at 90 °C and redissolved in methanol (300 µL). The methanol solution was transferred to a Wheaton v-vial charged with silver trifluoromethanesulfonate (20 mg, 40 µmol) and the methanol removed by heating under N_2_. The precursor **7** (16 mg, 40 µmol) was dissolved in dichloroethane (300 µL) and added to the vial by syringe. The sealed vial was kept at 60 °C for 20 min, after which the solvent was removed by heating under N_2_. Acetonitrile was added (400 µL) and the reaction mixture was filtered through a syringe filter (Puradisc 4mm, 0.2 µm) before injection into a radio-HPLC. The radiotracer was purified using a reverse-phase HPLC column (Phenomenex Luna C18(2) 100Å, 250 x 10 mm) at a flow rate of 3 mL/min, using water and acetonitrile as mobile phases. The following gradient was applied: 40-95% acetonitrile for 20 min; 95% acetonitrile for 5 min; 95-40% acetonitrile for 5 min. ^18^F-Pretomanid eluted at 13 min and was diluted in water to a total volume of 50 mL, trapped into a preactivated Sep-Pak C-18 light cartridge, and eluted with ethanol (1 mL) into a vial containing dimethyl sulfoxide (30-50 µL). The ethanol was evaporated under N_2_ and the radiotracer was formulated in 10% sulfobutylether-β-cyclodextrin, containing less than 10% dimethylsulfoxide. ^18^F-Pretomanid was obtained in >95% radiochemical purity and 2-8% radiochemical yield (n.d.c.) from start of synthesis to formulation.

*Automated cGMP synthesis:* Aqueous ^18^F-fluoride (1.8 mL) was trapped on a Chromafix 30-PS-HCO3 anion exchange cartridge and extracted with a solution of potassium bicarbonate (2.1 mg, 21 µmol) and Kryptofix 2.2.2 (6.7 mg, 18 µmol) in a mixture of acetonitrile and water (1 mL, 1:1) into a reaction vial. The solution was dried under a constant stream of N_2_ at 110 °C, and azeotropic distillation was performed with acetonitrile (250 µL, twice). The vial was transferred from the thermal heating position to the microwave heating station where it was cooled to room temperature with air. The precursor **7** (5 mg, 14 µmol) was dissolved in dimethylformamide (500 µL) and added to the reaction vial which was then heated under microwave irradiation at 100 watts for 10 min. A maximum temperature of 120 °C was applied. The reaction was quenched with a mixture of acetonitrile and water (2 mL) before purification by preparative-HPLC. The radiotracer was purified using a reverse-phase HPLC column (XBridge C_18_ 10 µm, 150 x 10 mm) at a flow rate of 10 mL/min, using methanol (50%) and 0.1 M ammonium formate (50%) as mobile phase. ^18^F-Pretomanid eluted at 8.8 min and was diluted in water to a total volume of 50 mL, trapped into a preactivated Sep-Pak tC18 cartridge, and eluted with ethanol (1 mL) followed by 0.9% sodium chloride (10 mL), via a sterilizing filter (0.22 µm), into a sterile vial containing 0.9% sodium chloride (4 mL). ^18^F-Pretomanid was obtained in >95% radiochemical purity and 5.7 ± 0.3% n.d.c. yield. The specific activity was measured as 68 ± 2 GBq/µmol (*n* = 3).

***In vitro* characterization of ^18^F-pretomanid**

*Calculation of LogD:* The LogD_7.4_ of ^18^F-pretomanid was determined by a partition method between octanol and PBS at pH 7.4. 1-octanol was pre-saturated with PBS before use. ^18^F-Pretomanid (1 µL, < 0.1 MBq) was added to a mixture of PBS (200 µL) and 1-octanol (200 µL) in a 1.5 mL Eppendorf vial (*n* = 6). The mixture was vigorously agitated at room temperature for 5 min and then centrifuged at 3000 g for 10 min. An aliquot (100 µL) from each layer was drawn for measurement in a gamma counter as counts per minute (CPM). The LogD_oct/PBS_ was calculated as follows:

LogD_oct/PBS_ = log [(CPM_1-octanol_ – CPM_1-octanol blank_) / (CPM_PBS_ – CPM_PBS blank_)]

*Serum stability and protein binding:* ^18^F-Pretomanid (10 µL, < 1 MBq) was added to serum (1 mL) and incubated at 37 °C for 3 h. An aliquot (200 µL) was removed at 10 min, 1 h, and 3 h time points. Serum stability was measured by radio-TLC. Proteins were separated out using a centrifugal filter (Amicon 10 k). The filter tubes were centrifuged at 13,000 RPM for 10 min, washed with PBS and centrifuged again. The filter was removed and the radioactivity in the filter and filtrate was measured by gamma counting to give separate counts per minute (CPM) for each component. The percentage of protein binding was calculated as follows:

% protein binding = CPM_filter_ / (CPM_filter_ + CPM_filtrate_)

*Red blood cell (RBC) partition coefficient:* ^18^F-Pretomanid (1.5 ± 0.6 MBq) was injected into mice (n=3) intravenously. Mice were euthanized 30 min later and blood was drawn into EDTA tubes (BD Microtainer, Fisher Scientific). The amount of ^18^F-pretomanid in each sample was quantified as counts per minute (CPM) by gamma counting separately. RBC partition coefficient was calculated as follows:

RBC partition coefficient = CPM_RBC_ / (CPM_RBC_ + CPM_Plasma_)

**Blood to plasma correction for imaging analysis**

PET blood (PET_blood_) data were corrected to PET plasma (PET_plasma_) data to enable tissue/plasma ratio calculations for comparison with mass spectrometry results from plasma. Standard hematocrit (Hct) in human subjects was 45% based on standard male and female ranges (*36*) and standard Hct in animal studies were based on published data for each animal model (*34, 35*). RBC partition coefficient was based on published literature (*37*) and results from experiments with radiolabeled-pretomanid (**table S5**). PET_plasma_ was converted from PET_blood_ using the following formula in human studies:

PET_plasma_ = PET_blood_ × [(1 – [RBC partition coefficient × Hct_subject_ / Hct_standard_]) / (1 – Hct_subject_)]

The following formula was used in animal studies:

PET_plasma_ = PET_blood_ × [(1 – RBC partition coefficient) / (1 – Hct_standard_)]

**FIGURES**


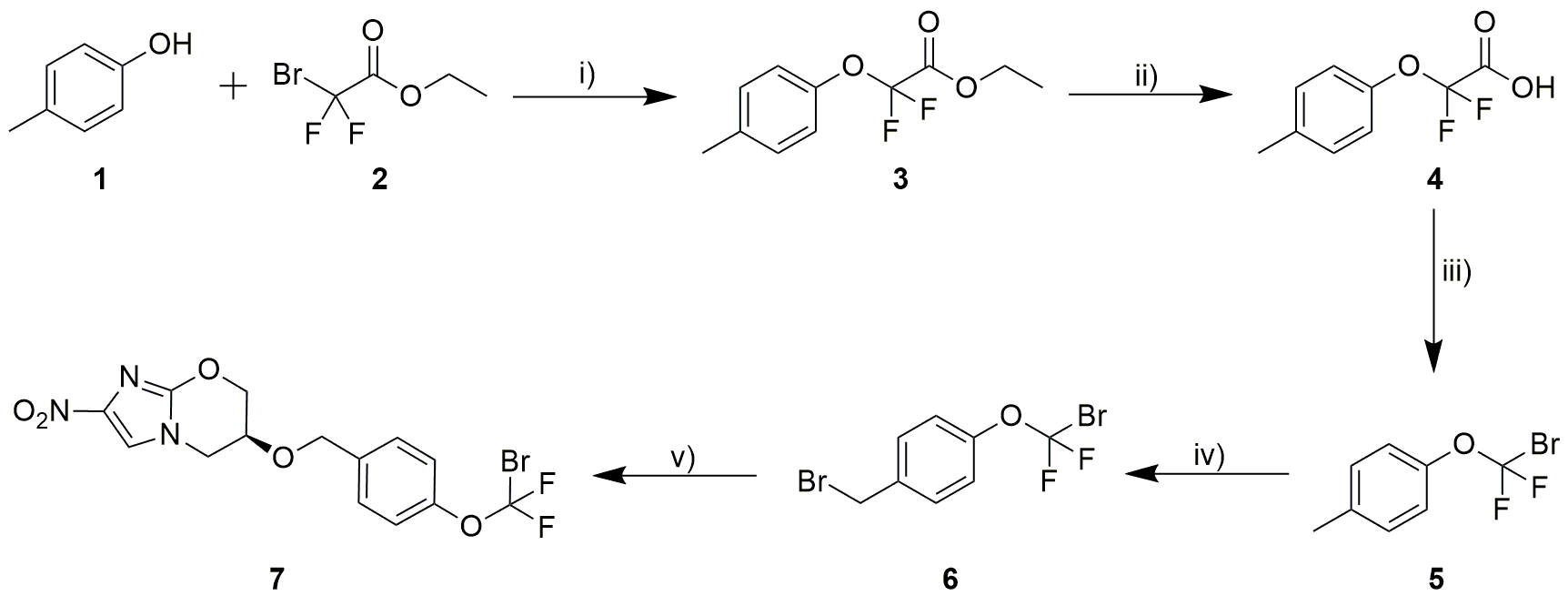


**Fig. S1. Precursor synthesis.** Precursor synthesis and radiolabeling was performed using the following reagents and conditions: i) NaH, DMF, 10 °C to room temperature (r.t.); ii) NaOH, acetonitrile, r.t., 5 h; iii) (i) DMF, DCM, oxayl chloride, 0 °C to r.t., 3 h, (ii) bromochloromethane, dimethylaminopyridine, 2-mercaptopyridine *N*-oxide sodium salt, reflux, 2 h; iv) carbon tetrachloride, *N*-bromosuccinimide, benzoyl peroxide, reflux, 5 h; v) (*S*)-2-nitro-6,7-dihydro-5H-imidazo[2,1-b][1,3]oxazin-6-ol, DMF, NaH, r.t., 3 h.

**
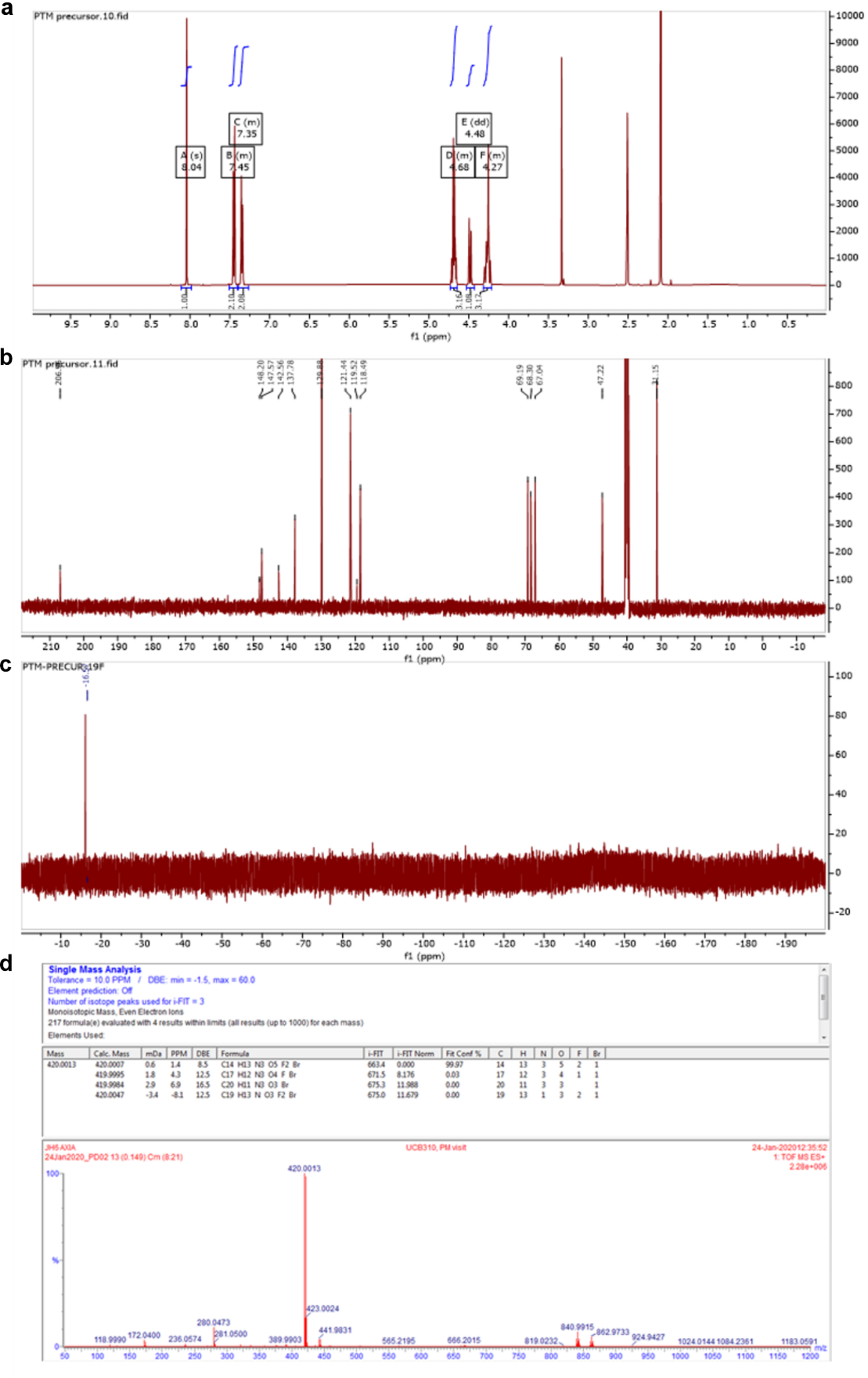
**

**Fig. S2. Structural determination of ^18^F-pretomanid precursor 7. a** ^1^H NMR. **b** ^13^C NMR. **c** ^19^F NMR. **d** Mass spectrum.


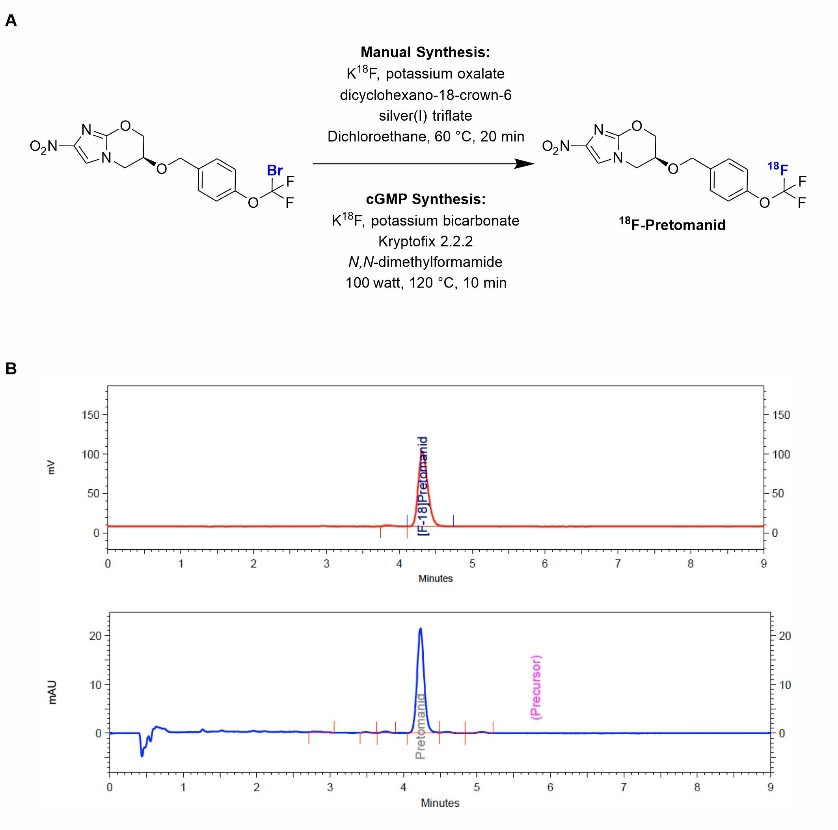


**Fig. S3. HPLC** **chromatograms of purified ^18^F-pretomanid.** Representative chromatograms of final product solution in the radio-HPLC trace (top red) showing ^18^F-pretomanid with >95% purity corresponding to the single peak for pretomanid in the UV trace at 328 nm (bottom blue).


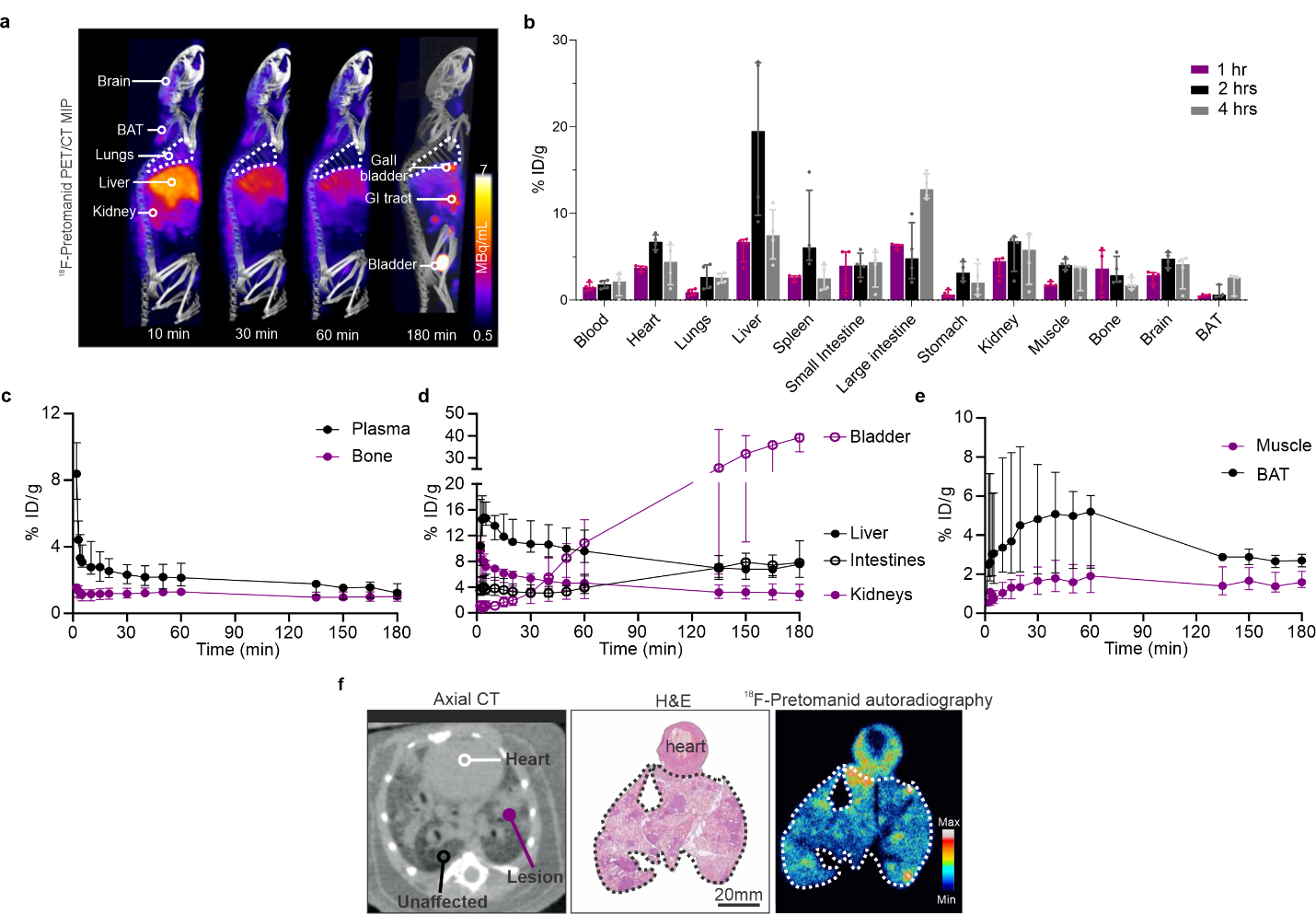
**Fig. S4. Validation of ^18^F-pretomanid in the mouse model of pulmonary TB** **a** ^18^F-Pretomanid PET/CT maximum intensity projections (MIP) at 10, 30, 60 and 180 min post-injection showing the whole-body distribution in a mouse with pulmonary TB. **b** Post-mortem biodistribution of ^18^F-pretomanid at 1, 2, and 4 h post-injection. **c** PET-derived ^18^F-pretomanid time-activity curves (TACs) from 0 to 180 min in plasma and bone. TAC represented as percentage of injected dose per gram (% ID/g) of tissue over time. **d** TAC from 0 to 180 min in liver, intestines, kidneys, and bladder. **e** TAC in brown adipose tissue (BAT) and muscle. **f** Axial CT scan and coronal histopathology and autoradiography of the same mouse showed lung lesions (e.g., granulomas, pneumonias), unaffected lung, and heart. The lungs are outlined by white and black dotted lines and autoradiography shows heterogeneous distribution of ^18^F-pretomanid. H&E=hematoxylin and eosin. *n* = 4 mice. PET studies are based on microdoses (ng-µg) administered intravenously. Data are represented as median ± interquartile range (IQR). Source data are provided as a Source Data File.

**
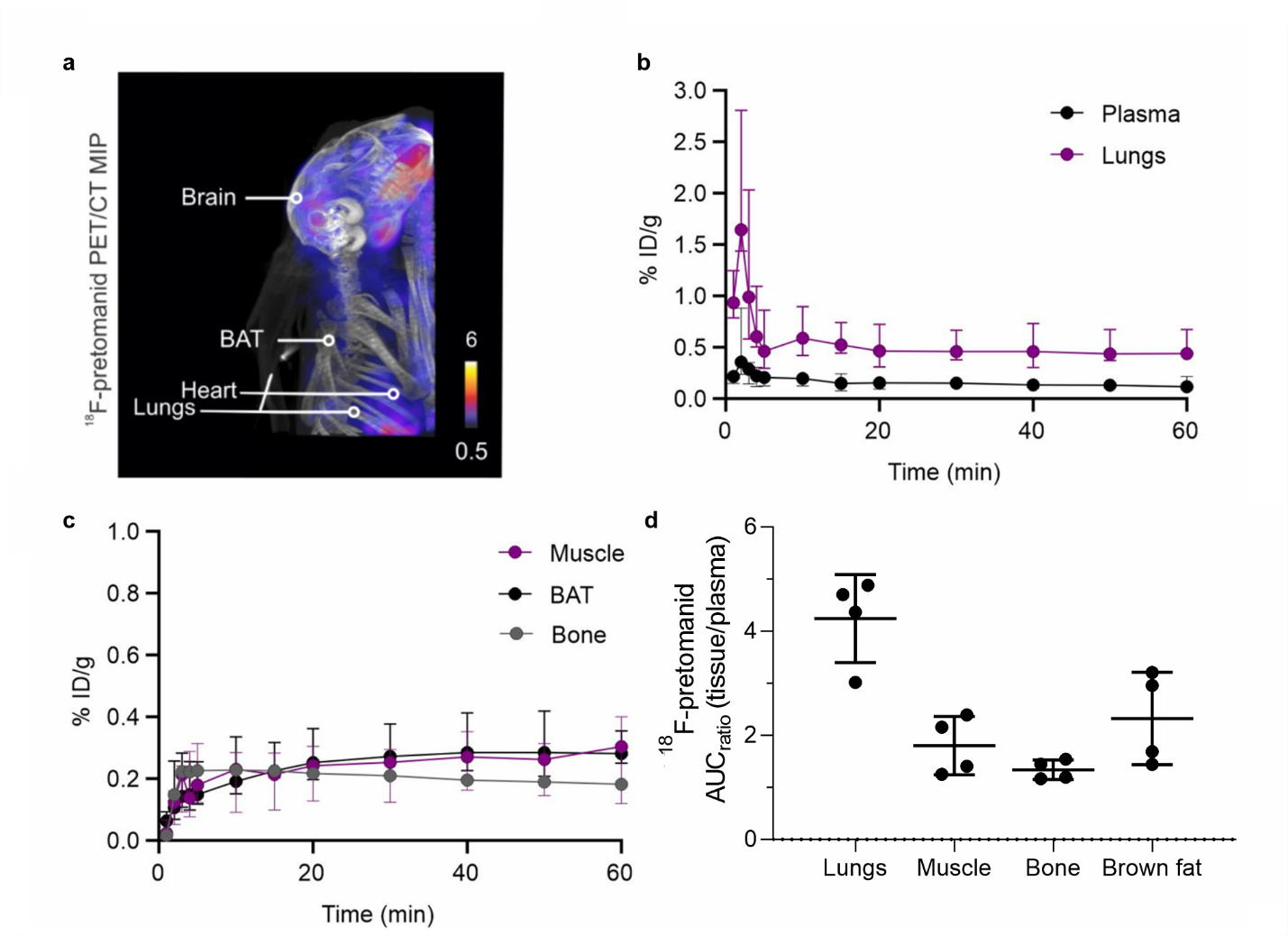
Fig. S5.** **^18^F-Pretomanid PET/CT imaging in the rabbit model of TB meningitis. a** ^18^F-Pretomanid PET/computed tomography (CT) maximum intensity projection (MIP) image in a rabbit with TB meningitis. **b** PET-derived ^18^F-pretomanid time-activity curves (TACs) from 0 to 60 min in plasma and lungs. **c** PET-derived ^18^F-pretomanid TACs from 0 to 60 min in muscle, brown adipose tissue (BAT) and bone. **d** ^18^F-Pretomanid AUC_3-60_ ratios (tissue/plasma). ^18^F-Pretomanid upper body biodistribution in the rabbits is similar to what is noted in the mouse studies. *n* = 4 rabbits; one VOI per tissue. PET studies are based on microdoses (ng-µg) administered intravenously. Data are represented as median ± IQR. Source data are provided as a Source Data file.

**
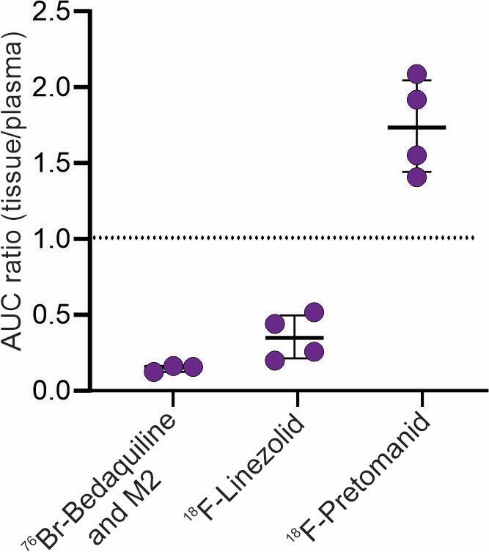
**

**Fig. S6.** **PET-derived brain exposures in mice.** PET-derived AUC ratios (brain/plasma) for ^76^Br-bedaquiline (including M2) AUC_0-48hours_* (*n* = 3), ^18^F-linezolid AUC_0-60min_* (*n* = 4), and ^18^F-pretomanid AUC_3-60min_ (*n* = 4) in healthy mice. *Levels for ^76^Br-bedaquiline and ^18^F-linezolid were derived from previously published studies (references 18 and 25). PET studies are based on microdoses (ng-µg) administered intravenously. Data are represented as median ± IQR. Source data are provided as a Source Data file.

**
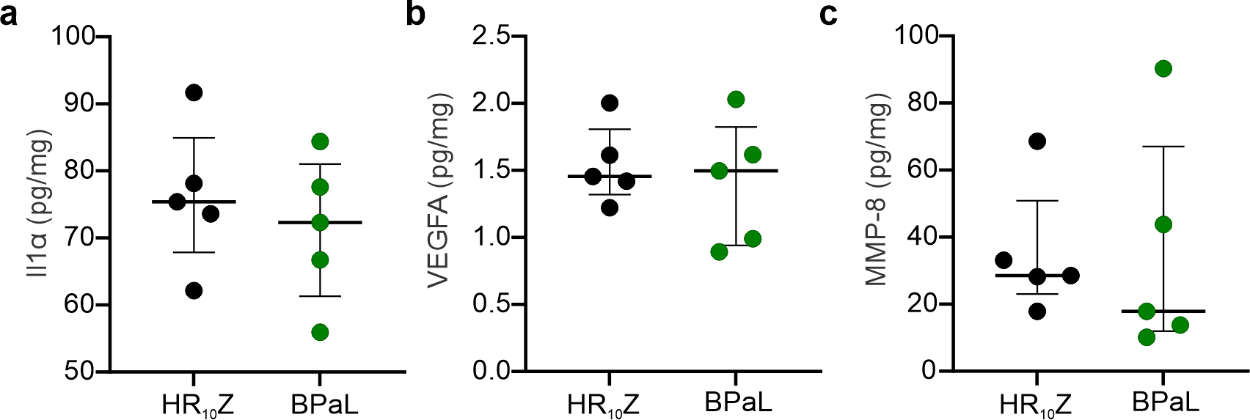
**

**Fig. S7. Cytokines in brain lysates in the mouse model of TB meningitis. a** IL1α, **b** VEGFA, **c** MMP-8 concentration from brain lysates (*n* = 5). Data are represented as median ± IQ and statistical comparisons were made using two-tailed Mann-Whitney-Wilcoxon test. Source data are provided as a Source Data file.

**
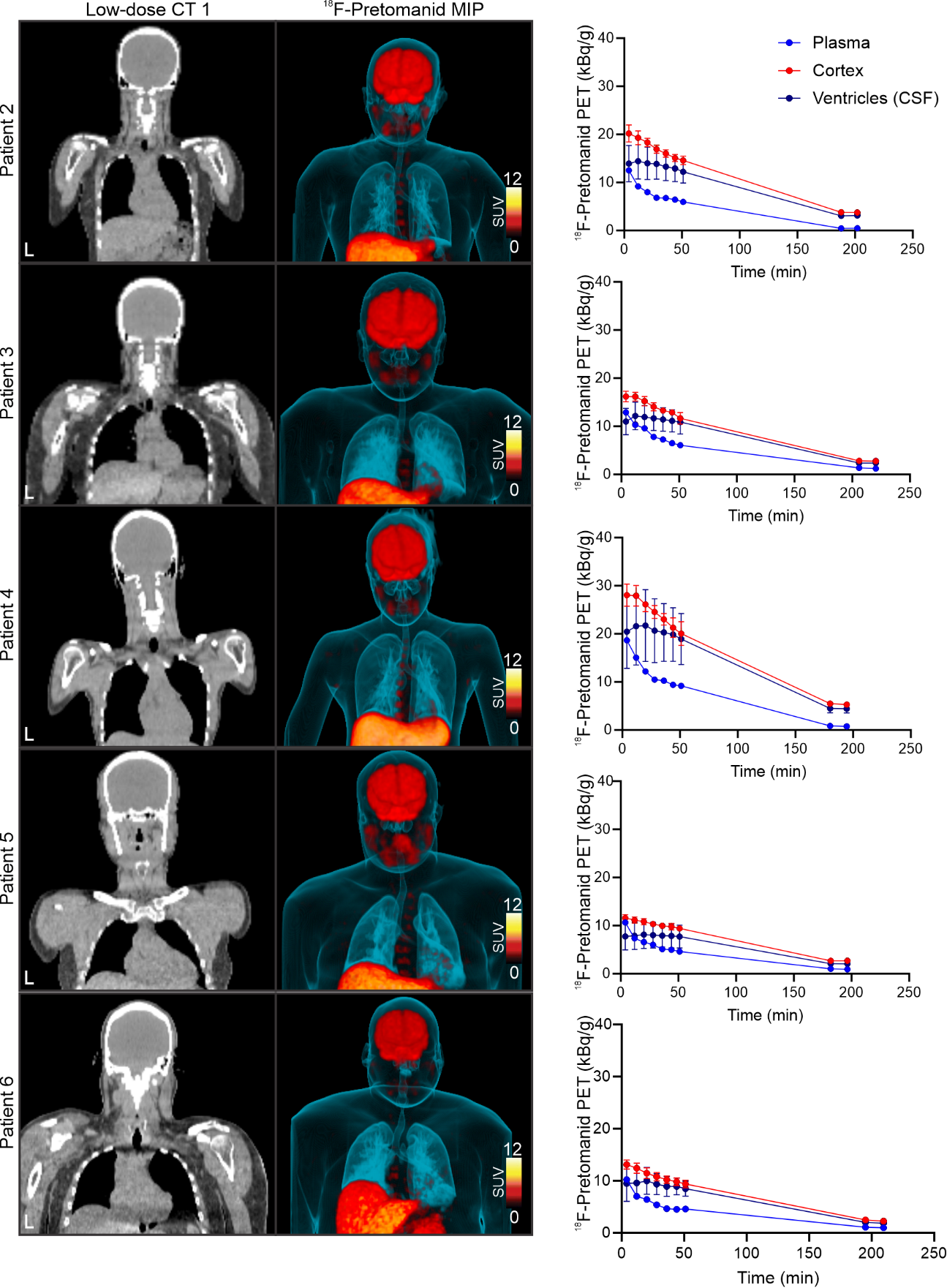
**

**Fig. S8. First-in-human ^18^F-pretomanid PET/CT studies.** Coronal CT, coronal maximum-intensity projection (MIP) images, and time-activity curves for the six subjects. Subject 1 is presented in Fig. 5. PET studies are based on microdoses (ng-µg) administered intravenously. SUV = standard uptake values. Data are represented as median ± IQR. Source data are provided as a Source Data file.

**Tables**

| **Plasma species** | **% Stability** | | | **% Protein binding** | | |
| --- | --- | --- | --- | --- | --- | --- |
|  | **10 min** | **60 min** | **180 min** | **10 min** | **60 min** | **180 min** |
| Human | 94 ± 11 | 94 ± 5 | 90 ± 3 | 75 ± 1 | 75 ± 1 | 77 ± 1 |
| Rabbit | 98 ± 4 | 96 ± 6 | 92 ± 1 | 78 ± 1 | 78 ± 1 | 80 ± 1 |
| Rabbit (infected) | 100 ± 0 | 91 ± 13 | 91 ± 0 | 80 ± 1 | 81 ± 0 | 83 ± 1 |
| Mouse | 100 ± 0 | 100 ± 0 | 100 ± 0 | 76 ± 2 | 75 ± 2 | 80 ± 1 |
| Mouse (infected) | 100 ± 0 | 100 ± 0 | 96 ± 6 | 74 ± 0 | 74 ± 2 | 78 ± 0 |

**Table S1.** **Serum stability and protein binding of ^18^F-pretomanid.** Data are represented as mean ± standard deviation (SD). *n* = 3 biological replicates per group. Source data are provided as a Source Data file.

|  | **Week 2** | | | **Week 6** | | |
| --- | --- | --- | --- | --- | --- | --- |
| **Regimen** | **CFU(Brain)/g (Log10) (mean)** | **SD** | **Log_10_ reduction** | **CFU(Brain)/g (Log10) (mean)** | **SD** | **Log_10_ reduction** |
| Untreated | 6.73 | 0.32 | - |  |  |  |
| HR_10_Z | 4.34 | 0.16 | 2.39 | 3.21 | 0.19 | 3.52 |
| BPaL | 5.25 | 0.28 | 1.48 | 4.86 | 0.19 | 1.89 |

**Table S2.** **Brain bacterial burden in the mouse model of TB meningitis.** Bacterial burden (log_10_ CFU) at week 2 and week 6 after initiation of treatment (*n* = 5-10 per animals per group in the treatment arms and 4 animals in the untreated group).

| **Drug** | **Single dose** | | | | | **Two-week treatment** | | |
| --- | --- | --- | --- | --- | --- | --- | --- | --- |
|  | **Plasma median µg/mL (IQR)** | **Brain median µg/mL (IQR)** | **CSF median**  **µg/mL (IQR)** | **Brain / plasma**  **median (IQR)** | **CSF/plasma**  **median (IQR)** | **Plasma median µg/mL (IQR)** | **Brain median µg/mL (IQR)** | **Brain/plasma**  **median (IQR)** |
| **Pretomanid** | 5.42  (3.58-12.17) | 12.26  (10.90-16-47) | 5.42  (4.1-7.0) | 2.56  (1.2-3.59) | 0.05  (0.018-0.14) | 9.09  (7.51-11.08) | 31.04  (24.09-35.52) | 3.39  (3.17-3.60) |
| **Linezolid** | 22.98  (15.25-33.0) | 4.44  (2.71-7.93) | 0.38  (0.1-0.76) | 0.16  (0.12-0.28) | 0.24  (0.27-0.31) | 30.36  (25.18-41.49) | 5.76  (4.6-8.12) | 0.19  (0.17-0.20) |
| **Bedaquiline** | 0.73  (0.34-1.19) | 0.02  (0.00-0.12) | 0.00  (0.00-0.00)* | 0.015  (0.00-0.16) | 0.00  (0.00-0.00) | 0.89  (0.42-1.19) | 0.05  (0.00-0.19) | 0.07  (0.01-0.13) |
| **M2** | 0.32  (0.14-0.77) | 0.08  (0.04-0.23) | 0.00  (0.00-0.00)* | 0.08  (0.00-0.36) | 0.00  (0.00-0.00) | 1.87  (1.36-2.28) | 0.42  (0.23-1.61) | 0.21  (0.16-0.51) |

**Table S3.** **Brain and CSF drug concentrations in TB meningitis mouse model.** Concentration of pretomanid, linezolid, bedaquiline, and bedaquiline metabolite (M2) in *M. tuberculosis*-infected mouse brains following a single dose and two weeks of treatment (10 doses). Tissues were collected at 4, 0.5, and 4 h for pretomanid, linezolid and bedaquiline, respectively. Data represented as median (IQR). Below limit of detection*

| **ID** | **Gender** | **Age (years)** | **Weight (kg)** | **BMI (kg/m^2^)** | **^18^F-Pretomanid** | | **Hematocrit (%)** | **Albumin (g/dL)** |
| --- | --- | --- | --- | --- | --- | --- | --- | --- |
|  |  |  |  |  | **Dose (MBq)** | **Specific activity (GBq/µmol)** |  |  |
| 1 | M | 28 | 84 | 27.5 | 355.2 | 38.7 | 43 | 4.5 |
| 2 | F | 20 | 68 | 26.6 | 362.6 | 41.7 | 40 | 4.6 |
| 3 | F | 29 | 75 | 28.2 | 358.9 | 23.6 | 39.3 | 4.8 |
| 4 | F | 24 | 48 | 18.3 | 358.9 | 40.7 | 38.7 | 4.7 |
| 5 | M | 53 | 98 | 31.5 | 362.6 | 62.7 | 41.5 | 4.3 |
| 6 | M | 35 | 116 | 37.8 | 358.5 | 34.2 | 43.7 | 4.6 |

**Table S4. First-in-human ^18^F-pretomanid PET studies.** BMI=body mass index, M=male, F=female.

| **Mice** | **RBC/blood** | **RBC/plasma** |
| --- | --- | --- |
| M1 | 0.48 | 2.5 |
| M2 | 0.71 | 2.5 |
| M3 | 0.64 | 1.9 |
| Average ±SD | 0.61 ± 0.12 | 2.3 ± 0.4 |

**Table S5. RBC partition coefficient for ^18^F-pretomanid.**
