# Supplementary material for: Dynamic 18F-Pretomanid PET imaging in animal models of TB meningitis and human studies
Source: Nat Commun. 2022 Dec 29;13:7974. doi: 10.1038/s41467-022-35730-3 (PMC9800570; doi:10.1038/s41467-022-35730-3)
Supplement: Supplementary file 3 — Reporting Summary [file 41467_2022_35730_MOESM3_ESM.pdf]

nature portfolio

Corresponding author(s): Sanjay K. Jain

Last updated by author(s): Dec 14, 2022

Reporting Summary

Nature Portfolio wishes to improve the reproducibility of the work that we publish. This form provides structure for consistency and transparency in reporting. For further information on Nature Portfolio policies, see our Editorial Policies and the Editorial Policy Checklist.

Statistics

For all statistical analyses, confirm that the following items are present in the figure legend, table legend, main text, or Methods section.

n/a	Confirmed	
☐	☒	The exact sample size (*n*) for each experimental group/condition, given as a discrete number and unit of measurement
☐	☒	A statement on whether measurements were taken from distinct samples or whether the same sample was measured repeatedly
☐	☒	The statistical test(s) used AND whether they are one- or two-sided<br>*Only common tests should be described solely by name; describe more complex techniques in the Methods section.*
☐	☒	A description of all covariates tested
☐	☒	A description of any assumptions or corrections, such as tests of normality and adjustment for multiple comparisons
☐	☒	A full description of the statistical parameters including central tendency (e.g. means) or other basic estimates (e.g. regression coefficient) AND variation (e.g. standard deviation) or associated estimates of uncertainty (e.g. confidence intervals)
☐	☒	For null hypothesis testing, the test statistic (e.g. *F*, *t*, *r*) with confidence intervals, effect sizes, degrees of freedom and *P* value noted<br>*Give P values as exact values whenever suitable.*
☒	☐	For Bayesian analysis, information on the choice of priors and Markov chain Monte Carlo settings
☒	☐	For hierarchical and complex designs, identification of the appropriate level for tests and full reporting of outcomes
☒	☐	Estimates of effect sizes (e.g. Cohen's *d*, Pearson's *r*), indicating how they were calculated

Our web collection on statistics for biologists contains articles on many of the points above.

Software and code

Policy information about availability of computer code

Data collection	Excel 2022 (Microsoft)
Data analysis	Excel 2022 (Microsoft), Prism 8.1.0 (Graphpad), VivoQuant 2020 (Invicro), PMOD (3.402), OsiriX MD 11.0 DICOM Viewer (Pixmeo SARL), RStudio Version 1.2.

For manuscripts utilizing custom algorithms or software that are central to the research but not yet described in published literature, software must be made available to editors and reviewers. We strongly encourage code deposition in a community repository (e.g. GitHub). See the Nature Portfolio guidelines for submitting code & software for further information.

Data

Policy information about availability of data

All manuscripts must include a data availability statement. This statement should provide the following information, where applicable:

- Accession codes, unique identifiers, or web links for publicly available datasets
- A description of any restrictions on data availability
- For clinical datasets or third party data, please ensure that the statement adheres to our policy

All data from this study is available in the manuscript and supplementary information.

Human research participants

Policy information about studies involving human research participants and Sex and Gender in Research.

Reporting on sex and gender	While both sexes were enrolled, no sex-based analyses were performed, as these were initial safety studies.
Population characteristics	Demographic characteristics of each study subject is provided in Fig. S8 and table S4. Subjects were recruited from Maryland, USA.
Recruitment	Study subjects provided permission to be contacted by the research team who confirmed eligibility. Written informed consent was obtained from each subject and screening labs performed thereafter. On the day of imaging, a board-certified physician reviewed screening labs, performed a physical exam and confirmed eligibility criteria. All study subjects that agreed to participate were included in this study.
Ethics oversight	This study was approved by the Johns Hopkins University Institutional Review Board.

Note that full information on the approval of the study protocol must also be provided in the manuscript.

Field-specific reporting

Please select the one below that is the best fit for your research. If you are not sure, read the appropriate sections before making your selection.

☒ Life sciences ☐ Behavioural & social sciences ☐ Ecological, evolutionary & environmental sciences

For a reference copy of the document with all sections, see nature.com/documents/nr-reporting-summary-flat.pdf

Life sciences study design

All studies must disclose on these points even when the disclosure is negative.

Sample size	The first-in-human studies were observational, to assess the safety of 18F-pretomanid PET and determine its pharmacokinetics and biodistribution in humans. Therefore, no sample size calculations were performed.
Data exclusions	No data was excluded in this study.
Replication	All data are representative of two or more independent experiments. All attempts at replication were successful.
Randomization	No randomization was used for patients. Rabbits and mice were randomly assigned to the treatment and control groups. No differences between animals were observed before treatment.
Blinding	The investigators involved in this study were not blinded during data collection and/or analysis.

Reporting for specific materials, systems and methods

We require information from authors about some types of materials, experimental systems and methods used in many studies. Here, indicate whether each material, system or method listed is relevant to your study. If you are not sure if a list item applies to your research, read the appropriate section before selecting a response.

Materials & experimental systems

n/a	Involved in the study	
☒	☐	Antibodies
☒	☐	Eukaryotic cell lines
☒	☐	Palaeontology and archaeology
☐	☒	Animals and other organisms
☒	☐	Clinical data
☒	☐	Dual use research of concern

Methods

n/a	Involved in the study	
☒	☐	ChIP-seq
☒	☐	Flow cytometry
☒	☐	MRI-based neuroimaging

Animals and other research organisms

Policy information about studies involving animals; ARRIVE guidelines recommended for reporting animal research, and Sex and Gender in Research

Laboratory animals	C3HeB/FeJ mice, female, 4-6 weeks of age, and New Zealand White rabbits, male and female, 5-7 days of age. Additional information is provided in the methods section.
Wild animals	No wild animals were used in this study.
Reporting on sex	No sex-based analyses were performed.
Field-collected samples	No field-collected samples were used in this study.
Ethics oversight	The Johns Hopkins University Animal Care and Use Committee approved all the animal experiments in this study.

Note that full information on the approval of the study protocol must also be provided in the manuscript.
